# Persistence of decidual NK cells and KIR genotypes in healthy pregnant and preeclamptic women: a case-control study in the third trimester of gestation

**DOI:** 10.1186/1477-7827-9-8

**Published:** 2011-01-19

**Authors:** Elly N Sánchez-Rodríguez, Sonia Nava-Salazar, C Adriana Mendoza-Rodríguez, Carlos Moran, Juan F Romero-Arauz, Enrique Ortega, Julio Granados, Alicia Cervantes-Peredo, Marco Cerbón

**Affiliations:** 1 Facultad de Química, Departamento de Biología, Universidad Nacional Autónoma de México. Ciudad Universitaria, Coyoacán 04510, México, D.F., México; 2 Servicio de Complicaciones Hipertensivas, UMAE de Ginecología y Obstetricia "Luis Castelazo Ayala", Instituto Mexicano del Seguro Social, México, D.F., México; 3 Departamento de Inmunología, Instituto de Investigaciones Biomédicas, UNAM, Mexico, D.F., México; 4 Departamento de Transplantes, Instituto Nacional de Ciencias Médicas y de la Nutrición "Salvador Zubirán", México, D.F., México; 5 Servicio de Genética, Hospital General de México/Facultad de Medicina, UNAM, México, D.F., México

## Abstract

**Background:**

Natural Killer (NK) cells are the most abundant lymphocytes in the decidua during early gestation. The interactions of NK cells with the extravillous cytotrophoblast have been associated with a normal spiral artery remodeling process, an essential event for a successful pregnancy. Recent data indicate that alterations in the amount of decidual NK (dNK) cells contribute to the development of preeclampsia (PE). Moreover, genetic studies suggest that Killer Immunoglobulin-like Receptors (KIR) expressed in dNK cells influence the susceptibility to PE. Although dNK cells have been well characterized during early pregnancy, they have been scarcely studied in the third trimester of gestation. The aim of this work was to characterize dNK cells at the last trimester of gestation and to analyze the KIR genotype of healthy and PE women.

**Methods:**

Decidual samples were obtained during Caesarean section from control (n = 10) and PE (n = 9) women. Flow cytometric analysis of CD3, CD56, CD16 and CD9 was used to characterize and quantify dNK cells in both groups. Cell surface markers from decidual leukocytes were compared with PBMC from healthy donors.

KIR genotyping was performed in genomic DNA (control, n = 86; PE, n = 90) using *PCR-SSP.*

**Results:**

The results indicate that dNK cells persist throughout pregnancy. They represented 20% of total leukocytes in control and PE groups, and they expressed the same cell surface markers (CD3^-^, CD56^+^, CD16^- ^and CD9^+^) as dNK in the first trimester of gestation. There were no significant differences in the percentage of dNK cells between control and PE groups. The analysis of KIR gene frequencies and genotypes was not statistically different between control and PE groups. The ratio of activating to inhibitory genes indicated that the overall inhibitory balance (0.2-0.5) was more frequent in the PE group (control, 31.3% *vs *PE, 45.5%), and the activating balance (0.6-1.1) was more frequent in the control group (control, 68.6% *vs *PE, 54.4%). However this difference was not significant.

**Conclusion:**

We demonstrated the persistence of dNK cells in PE and control women at the third trimester of pregnancy; these dNK cells had a similar phenotype to those found during early pregnancy. The predominance of a KIR inhibitory balance in the PE group could be associated to the physiopathology of PE.

## Background

Extravillous trophoblast invasion is a hallmark of normal pregnancy. One of the main mechanisms that contribute to normal spiral artery remodeling is the interaction between decidual leukocytes and the extravillous cytotrophoblast. Dysregulation of these interactions is associated with shallow endovascular cytotrophoblast invasion of the spiral arteries, which is a key feature in the pathogenesis of preeclampsia (PE) [1].

During normal early pregnancy, Natural Killer (NK) cells are the most abundant population of leukocytes in the decidua. It has been reported that NK cells represent 70% of the total decidual leukocyte population in the first trimester of gestation [2,3]. Some authors propose that this population is practically absent at the end of pregnancy [2,4], but other researchers have identified and isolated decidual NK cells (dNK) during normal term pregnancies [5-9].

It is well accepted that dNK cells differ from peripheral blood NK cells (pNK) in terms of their gene expression pattern, cell surface markers [10] and functions [11]. Based on their cell surface markers, pNK cells are divided into two subsets: CD56^dim ^CD16^+ ^(highly cytotoxic cells) and CD56^bright ^CD16^- ^(high cytokine secretors) [12]. During early pregnancy, dNK cells have been characterized as CD56^bright ^CD16^-^, as well as by the expression of some exclusive proteins, e.g., Killer cell Immunoglobulin like Receptors (KIR), CD9, CD151, CD53 and α4β7 integrin [10,13,14].

Recently, Hanna *et al *found that dNK cells but not pNK cells, mediate angiogenesis and fetal trophoblast invasion through the release of chemokines and proangiogenic factors, including vascular endothelial growth factor (VEGF) and placental growth factor (PLGF) [11]. In samples of decidua basalis in the earliest stages of artery remodeling, Smith *et al *found dNK immunopositive cells for matrix metalloproteinase 7 and 9, suggesting their participation in vascular remodeling in a trophoblast independent stage [15].

Less evidence is available regarding the proportion, cell surface markers and functions of dNK cells from patients with PE, and the data are still controversial. Some authors have found an increase in the number of CD56^+ ^cells in the decidua of women with PE compared to normal term pregnant women [16,17], while others have found a decrease [18-20] or no differences in the number of cells [21]. In this study, we analyzed NK cells from the decidua of PE patients and normal term pregnant women.

Several functions of NK cells depend on a finely tuned regulation by inhibitory and activating receptors. The KIR family is one of the most important groups of receptors expressed in NK cells, and HLA (histocompatibility leukocyte antigens) are their foremost ligands. The KIR family contains inhibitory (L) and activating (S) receptors encoded by highly polymorphic loci. The number and type of genes in these loci vary among individuals and haplotypes. Group A haplotype contains only one stimulatory KIR gene (*2DS4*), whereas group B haplotype contains various combinations of stimulatory genes [22-24].

There is evidence about the participation of KIR in the development of PE. The immune response in the decidua greatly depends on the receptor-ligand recognition of maternal KIR genotypes and their fetal ligands during the critical stages of uterine arterial remodeling. In a Caucasian population it has been reported that the KIR inhibitory genotype *AA *from pregnant women in combination with *HLA-C2 *ligands in their babies, specifically the presence of *KIR2DL1*, is associated with an increased prevalence of PE [25]. In the present study we analyzed the KIR genotype in normal and PE Mexican women.

## Methods

### Patients and biological samples

For the analysis of decidual leukocytes, decidual tissue samples were obtained by scraping off the mucosal surface of the uterine cavity by curettage during cesarean section, of women that had indicated or elective cesarean sections at the third trimester of gestation (>36 weeks of gestation; control, n = 10; PE group, n = 9). None of the subjects were in labor. Peripheral blood samples were obtained from pregnant women in the first trimester of gestation and from healthy non-pregnant women.

For the KIR genotyping study, maternal peripheral blood samples were obtained by venipunction of the forearm from eighty-six (n = 86) healthy pregnant women (control group) and ninety (n = 90) preeclamptic women (PE group).

This case-control study was performed with women recruited consecutively from the Hospital of Gynecology "Luis Castelazo" of the Mexican Social Security Institute, in Mexico City. For the PE group, the diagnosis criteria and classification were according to the American and Royal College of Obstetricians and Gynecologists [26,27] and the Mexican Ministry of Health guidelines [28]. Severe PE was defined as new-onset hypertension after the 20^th ^week of pregnancy; systolic pressure ≥160 mm Hg and/or diastolic ≥110 mm Hg, during a seven day period on two occasions at least 6 hours apart, while the patient is on bed rest; proteinuria ≥ 2 g in a 24 h urine specimen; two or more persistent data of vasospasm (e.g. oliguria of less than 500 mL in 24 h, seric creatinin ≥ 1.2 mg/dL, cerebral or visual disturbances, pulmonary edema, epigastric or right upper-quadrant pain, impaired liver function [AST ≥ 70/UI], thrombocytopenia [<100,000 mm^3^], cyanosis, intrauterine growth restriction in the babies).

The control group consisted of healthy pregnant women at the end of pregnancy, without any data or history of PE. Exclusion criteria involved patients with diabetes mellitus, gestational diabetes mellitus, renal diseases, chronic hypertension, infectious diseases, autoimmune diseases, and chronic diseases in general.

The Hospital Ethics Committee approved this study and written consent was obtained from all women.

### Decidual leukocytes isolation and Flow cytometry

In order to isolate leukocytes from the decidua and to characterize NK cells, tissue samples were processed as described by Rieger *et al *[19], and Arcuri *et al *[29], with minor modifications. Briefly, decidual tissues were carefully cleaned of visible blood clots, specimens were extensively washed (at least three times) in sterile PBS solution to eliminate any possible blood contamination; and were then trimmed, minced and digested with 0.1% (w/v) collagenase type IV (Sigma Chemical, St. Louis, MO, USA) and 0.02% (w/v) hyaluronidase type I-S (Sigma Chemical, St. Louis, MO, USA) in PBS for 1 h at 37°C under gentle agitation. The cell suspension was filtered through a 70 μm sieve (Becton Dickinson Labware, Franklin Lakes, NJ, USA), washed in PBS and layered on an equal volume of Ficoll Hypaque (Sigma Chemical, St. Louis, MO, USA) at room temperature for density gradient centrifugation (25 min at 600 g). Cells were collected from the interface, washed and suspended in RPMI 1640, supplemented with 10% FCS, 100 IU/mL penicillin, 100 IU/mL streptomycin and 0.25 μg/mL amphotericin B (Gibco BRL, Grand Island, NY, USA).

Cells were labeled by direct staining with monoclonal antibodies using single, double or triple color immunofluorescence staining: CD3 [HIT3a; FITC] and CD16 [DJ130c; FITC] from Santa Cruz (Santa Cruz, CA, USA); CD56 [N-CAM clone MEM 188; Phycoerythrin] and CD9 [OKT3; FITC] from e-Bioscience (San Diego, CA, USA). For three-color staining we used a third antibody for CD3 [clone SK7; PerCP] from Becton Dickinson (San Jose, CA, USA). 1 × 10^6 ^cells/mL in PBS/0.1% sodium azide (300 μL/sample) were incubated with 10 or 20 μL of the appropriate monoclonal antibody for 30 min at 4°C in the dark. After two washes with PBS/0.1% sodium azide and once with PBS, cells were resuspended and fixed with PBS containing 1% paraformaldehyde for 20 min at 4°C in the dark. After one wash with PBS, cells were resuspended in PBS and analyzed on a Becton Dickinson FACSCalibur Flow cytometer.

In order to characterize more closely the decidual leukocyte population isolated and to establish qualitative differences in the subset markers between decidual and peripheral NK cells, using two-color staining the percentage of CD56^+^CD9^+ ^cells was compared with the same region in peripheral blood mononuclear cells (PBMC). A further characterization was done using triple-color immunofluoresce staining, CD3^- ^cells were gated and the CD56^+^CD9^+ ^expression was analyzed in this gate to avoid CD3^+^CD56^+ ^contamination in the analysis of the CD9 marker.

PBMC were obtained from healthy pregnant and non-pregnant women, by Ficoll Hypaque (Sigma Chemical, St. Louis, MO, USA) gradient and stained using the procedure described above.

Data were analyzed using BD Cell Quest™Software (San Jose, CA, USA). The percentage of CD3^+ ^(mature T lymphocytes), CD56^+ ^and CD3^-^/CD56^+^CD16^-/+ ^(NK cells population) was established by delineating regions around the lymphocyte cluster.

### KIR genotyping

DNA was extracted from peripheral blood of PE and control groups using a standard salting out procedure. KIR genes were genotyped by the presence or absence of each gene locus, using a low-resolution *PCR-SSP *commercial typing kit, following the manufacturers' instructions (Miltenyi Biotec, Bergisch Gladbach, Germany). We determined the KIR genotypes responsible for the inhibitory functions (*2DL1, 2DL2, 2DL3, 2DL4, 2DL5, 3DL1, 3DL2*, *3DL3*), the activating signals (*2DS1, 2DS2, 2DS3, 2DS4, 2DS5, 3DS1*) and the two pseudogenes (*2DP1 *and *3DP1*). The amplification products were electrophoresed on 2% agarose gels containing ethidium bromide, and visualized under ultraviolet light. The presence of each KIR gene was detected as a band of the expected size. Individuals were determined negative for a KIR gene when a band of the expected size was absent and the control band was present. For the assessment of a given genotype, *KIR2DS4 *full length (*ins*) and deletion (*del*) variants were assigned positive when either or both variants were detected; whereas when both variants were absent the subject was labeled as *2DS4 *negative.

### Statistics

The decidual leukocyte population was analyzed by Student's *t*-test. If the samples did not show a normal distribution, a non-parametric test was used (Mann Whitney's *U*-test). The *p *values lower than or equal to 0.05 were taken as significant.

Genotype frequency was determined by direct counting. Genotype frequency comparisons were made by a two-sided Fisher's exact test; when the *p *value was less than 0.05 we adopted a Bonferroni correction (*pc*) for multiple comparisons. The odds ratio (OR) and 95% confidence interval (CI) were calculated. The sample size required for an alpha of 0.05 and 80% of statistical power was calculated in each group.

All statistical analyses were performed in Prism 5 (GraphPad Software, Inc., La Jolla, CA, USA) for *Windows*.

## Results

### Flow cytometry of decidual leukocytes

**Figure 1:**
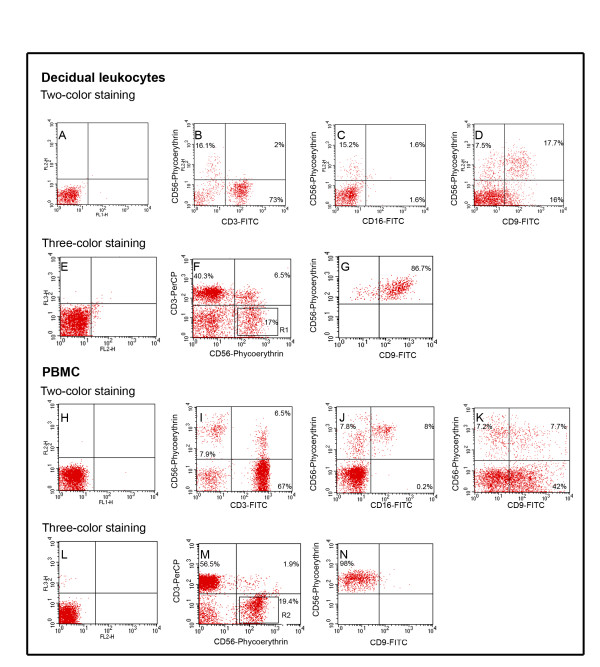
**Flow cytometry analysis of decidual leukocytes**. Comparison of typical subset-markers of NK cells (CD3^-^, CD56^+^, CD16^+/-^) and the non-typical dNK cells marker (CD9^+^). A-D) Representative FACS for two-color staining for decidual leukocytes. E-G) Representative FACS for three-color staining for decidual leukocytes. H-K) Representative FACS two-color staining for peripheral blood mononuclear cells (PBMC). L-M) Representative FACS three-color staining for PBMC. A, E, H, L) negative cells; B, I) upper left quadrants (UL): CD56^+ ^CD3^- ^cells; C, J) UL: CD56^+ ^CD16^- ^cells; upper right quadrants (UR): CD56^+ ^CD16^+ ^cells; D, K) UR: CD56^+^CD9^+ ^cells. F, M) R1 and R2, gate for CD3^- ^cells; G, N) CD56^+^CD9^+/- ^expression on CD3^- ^cells.

The cell surface markers and proportion of isolated leukocytes from third trimester decidual tissue are shown in Figure 1 and Table 1, respectively. Figure 1A, E, H and 1L show the negative control cell staining. The classic markers for NK cells, CD56^+^CD3^- ^(16.1% Figure 1B); and CD56^+^CD16^+/- ^(1.6% Figure 1C) in the decidua were compared with those obtained from the same subset of cells in PBMC (7.9% Figure 1I and 8% Figure 1J). The phenotype found in leukocytes from the decidua was similar to the CD56^+ ^CD16^- ^subpopulation in PBMC however, the CD56^+ ^CD16^+ ^subpopulation was not found in the decidua. Double positive cells were found for CD56 and CD9 markers in decidual leukocytes and PBMC, using two-color staining (Figure 1D, upper right quadrant and Figure 1K, upper right quadrant). However, when we used three-color staining (Figures 1E-G and 1L-N), 87% of the CD3^- ^CD56^+ ^subpopulation in the decidua (Figure 1F, R1) was CD9^+ ^(Figure 1G). In contrast, 98% of the same subpopulation in PBMC (Figure 1M, R2) was negative for the CD9 marker (Figure 1N).

**Table 1:** Proportion of decidual leukocytes analyzed by Flow cytometry

Decidual lymphocyte subset	Controls (n = 10)	PE (n = 9)	*p*
**CD3**^**+ **^**(%)**	54.15 ± 17.76	30.95 ± 15.01	**0.007 **^**a**^
**CD56**^**+ **^**(%)**	21.70 ± 10.35	23.24 ± 12.24	NS
**CD3**^**+ **^**CD56**^**+ **^**(%)**	2.34 ± 1.55	2.40 ± 2.05	NS
**CD3**^-^**CD56**^**+ **^**(%)**	20.66 ± 9.49	21.13 ± 12.79	NS
**CD56**^**+**^**CD16**^- ^**(%)**	19.09 ± 9.49	21.41 ± 12.36	NS
**CD56**^**+**^**CD16**^**+ **^**(%)**	1.65 ± 1.17	2.81 ± 4.53	NS
**CD56**^**+**^**CD9**^**+ **^**(%)**	15.68 ± 4.88	18.87 ± 8.7	NS

Comparison of the proportion of decidual leukocytes between healthy pregnant women (controls) and women with preeclampsia (PE) using two-color immunofluorescence.

Values are presented as mean ± standard deviation; NS, No significant difference; (%) percentage of cells bearing a particular set of subset markers.

^a ^*p *< 0.05

The classic markers for NK cells (CD3^-^CD56^+^) were detected in a similar percentage in the control and PE decidual tissue (20.66 ± 9.49% *vs *21.13 ± 12.79% respectively; *p *> 0.05) (Table 1). The subset-markers, CD56^+^CD16^-/+ ^and CD56^+^CD9^+ ^remained similar between both groups (*p *> 0.05) (Table 1).

Interestingly, significant differences in the proportion of CD3^+ ^cells between PE patients and the control group were found. A decreased percentage of CD3^+ ^cells in the PE group were observed as compared with the control group (30.95 ± 15.01% *vs *54.15 ± 17.76%, respectively; *p *= 0.007) (Table 1).

### KIR gene frequencies

To establish KIR gene frequencies, KIR genotyping was performed in women with PE and controls (Table 2). Framework genes (*2DL4 *and *3DL3)*, and the two pseudogenes (*2DP1, 3DP1*) were detected in 100% of women from both groups. The *3DL2, 2DL1, 3DL1*, and *2DL3 *genes were not found in all individuals (90.7-98.9%). The lowest frequency in both groups was for the *2DS3 *gene (<21%).

**Table 2:** Frequencies of KIR genes

KIR gene	Controls (n = 86)	PE (n = 90)
Inhibitory		
*2DL1*	96.5	98.9
^*a*^*2DL2*	61.6	43.3
*2DL3*	94.2	95.6
*2DL4*	100.0	100.0
*2DL5 all*	61.6	47.8
*2DL5 A*	59.3	50.0
^*b*^*2DL5 B*	64.0	48.9
*3DL1*	90.7	93.3
*3DL2*	97.7	95.6
*3DL3*	100.0	100.0
Activating		
*2DS1*	58.1	47.8
*2DS2*	48.8	36.7
*2DS3*	20.9	18.9
*2DS4 del*	65.4	54.1
*2DS4 ins*	75.3	76.5
*2DS5*	53.3	43.3
*3DS1*	52.3	48.9
Pseudogenes		
*2DP1*	100.0	100.0
*3DP1*	100.0	100.0

Values are presented as percentage.

^a ^*p *= 0.017, OR = 0.47, 95% CI = 0.26 to 0.86; *pc *> 0.05

^b ^*p *= 0.049, OR = 0.54, 95% CI = 0.23 to 0.98; *pc *> 0.05

The *p *values were corrected for 19 comparisons using the Bonferroni correction.

The frequencies of *KIR2DL2 *(control, 61.6% *vs *PE, 43.3%*; p *= 0.017, OR = 0.47, 95% CI = 0.26 to 0.86; *pc *> 0.05) and *2DL5 B *(control, 64.0% *vs *PE, 48.9%; *p *= 0.049, OR = 0.54, 95% CI = 0.23 to 0.98; *pc *> 0.05) were lower but not significantly different in the PE group as compared to control group (Table 2). No association for the activating KIR genes (*2DS1, 2DS2, 2DS3, 2DS4 del, 2DS4 ins, 2DS5 *and 3DS1) between groups was found. No significant differences were observed in any single KIR gene frequency after we adopted the Bonferroni correction.

### KIR genotype profiles

**Figure 2:**
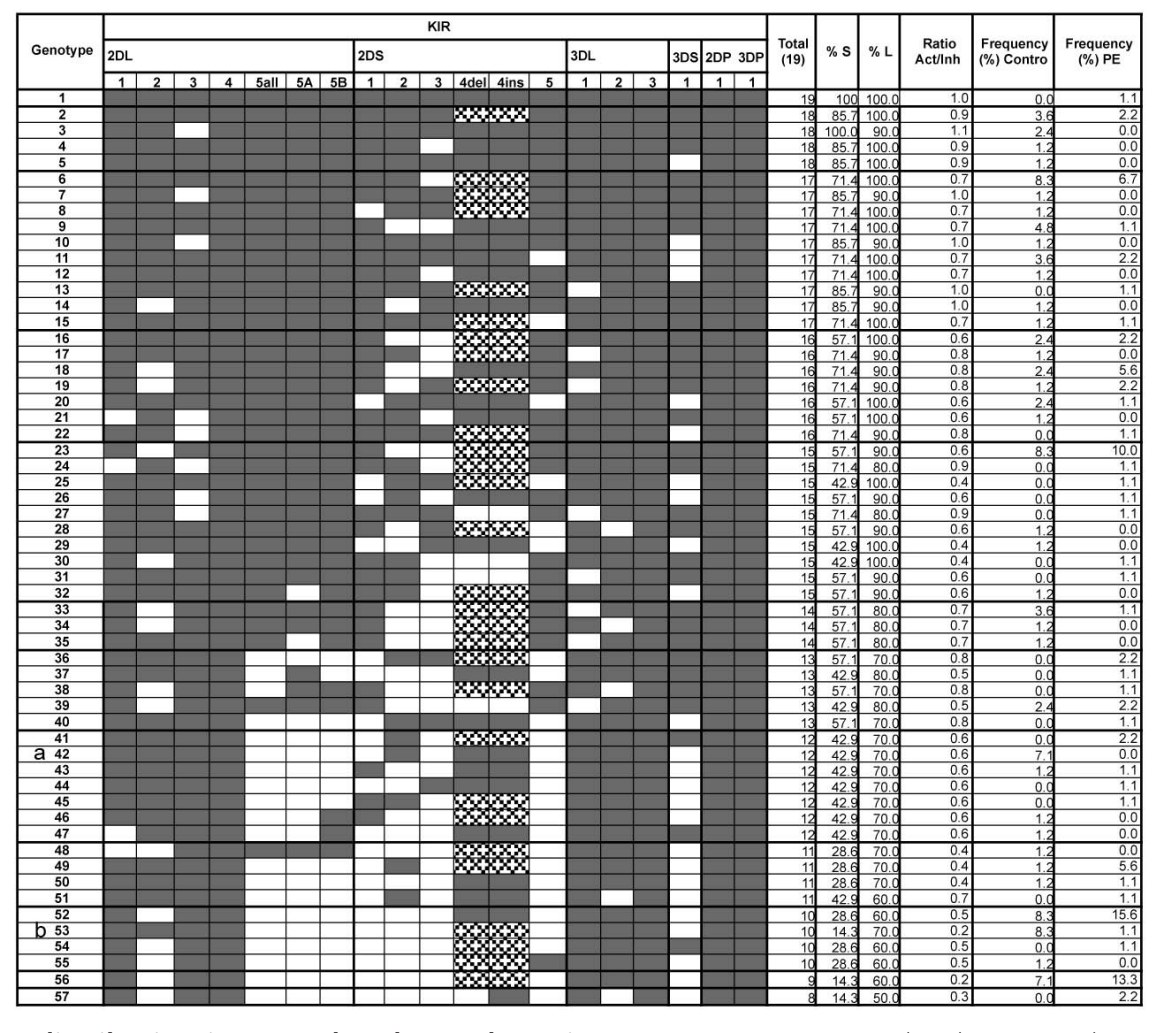
**KIR genotype distribution in control and preeclamptic women**. KIR genotype distribution and percentage (%) of women displaying each genotype in healthy women (Controls) and women with preeclampsia (PE). Shaded boxes represent the presence of the inhibitory genes (*2DL1, 2DL2, 2DL3, 2DL4, 2DL5all, 2DL5A, 2DL5B, 3DL1, 3DL2, 3DL3*), activating (*2DS1, 2DS2, 2DS3, 2DS4 del, 2DS4 ins, 2DS5, 3DS1*) and pseudogenes (*2DP1, 3DP1*). White boxes indicate the absence of KIR gene. Dark boxes indicate the presence of KIR gene. Dashed boxes indicate that the genotype was assigned when either *2DS4 ins *and *2DS4 del *variants were detected. The total number of KIR genes in each genotype is indicated, as well as the numbers of activating (S) and inhibitory (L) genes. Ratios of the activating to inhibitory genes are showed in the right column. The *p *values less than 0.05 were corrected for 57 comparisons using the Bonferroni correction. ^a ^*p *= 0.012, OR = 0.067, 95% IC 0.004 to 1.204; *pc *> 0.05 ^b ^*p *= 0.03, OR = 0.124, 95% IC 0.015 to 1.028; *pc *> 0.05

Fifty-seven combinations of KIR genes were found (Figure 2). The genotypes (G) with a lower number of KIR genes (G. 48 to G. 57) had at least one activating gene and six or seven inhibitory genes. Genotypes 23, 52 and 56 were detected in high frequencies (10% to 15.6%), whereas the rest of the genotypes were found in low frequencies (0%-8.3%).

Genotypes 42 and 53 were more prevalent but not significantly different in the control group as compared with the PE group (*pc *> 0.05); both genotypes were characterized by the presence of the *KIR2DL2 *gene. Genotype 52 (*KIR2DL2 *negative) was two fold more frequent in the PE group than in the control one, but this difference was not significant.

The ratios of activating to inhibitory (Act/Inh) genes ranged from 0.2 to 1.1. No significant differences were observed in the individual ratios between groups. In order to further analyze these data, we grouped the ratios into 2 range categories: 0.2 to 0.5 (more inhibitory range) and 0.6 to 1.1 (more activating range) (Table 3).

**Table 3:** Activating to inhibitory balance

Range Act/Inh	Frequency (%) Controls	Frequency (%) PE
	(n = 86)	(n = 90)
**Activating range: 0.6-1.1**	68.6	54.4
**Inhibitory range: 0.2-0.5**	31.3	45.5

Ratio numbers of activating to inhibitory (Act/Inh) KIR genes between controls and women with preeclampsia (PE). 0.2 to 0.5 (inhibitory range) and 0.6 to 1.1 (activating range).

*p *= 0.05, OR = 0.54, 95%CI 0.29 to 1.013

With this analysis we observed that the PE women fall more frequently (45.5%) into the inhibitory range category than controls women (31.3%) and the controls women fall more frequently (68.6%) in the activating range as compared to PE group (54.4%), however this difference was not significant (*p *= 0.05, OR = 0.54, 95% IC = 0.29 to 1.013).

## Discussion

Several studies indicate that dNK cells have a major role in implantation and placental development; alterations in their proportion and functions have been associated with PE [11,25,30]. There is growing evidence that the clinical features of PE are generated either by defective placentation during early pregnancy and/or by a distinct increase in the systemic inflammatory response in the second part of pregnancy [31,32].

There is agreement on the presence of NK cells in decidual tissue at the earliest stages of pregnancy, but there are conflicting reports about their presence at the third trimester of gestation [5-8,33,34]. In this study we found a high percentage of CD3^-^CD56^+ ^cells in the decidua of women at the third trimester of pregnancy, in agreement with previous investigations in which NK cells have been found and isolated from deciduas at term [5-8]. This is the first demonstration that this population shares the same phenotype as the dNK cells detected in the first trimester of gestation (CD56^+ ^CD16^- ^and CD9^+^) [14].

The CD9 marker has been proposed as a specific marker for dNK cells, it has been observed exclusively expressed in NK cells isolated from decidua in early gestation but not in pNK cells [10]. In placenta, it has been detected by immunohistochemistry on extravillous trophoblast cell columns of first trimester, but not in the third trimester [35]. We found in the CD3^- ^gate, double positive cells for CD56 and CD9 markers in leukocytes from decidua and not in PBMC, suggesting that the CD3^-^CD56^+ ^CD9^+ ^subpopulation found in the third trimester of gestation in the decidua, is similar to that found in early pregnancy. However, we also observed CD9 positive cells in all isolated PBMC when we used two-color staining flow cytometry and since not all CD56^+ ^cells in PBMC lack expression of CD3, there will be a possibility that the CD56^+^CD9^+ ^cells were CD3^+ ^(NKT). The expression of CD9 in peripheral leukocytes has been reported during infectious conditions [36]. This observation is clarified when we used three-color staining flow cytometric analysis, because all NK cells in PBMC were negative for CD9.

The ligand for murine CD9 is the pregnancy specific glycoprotein (PSG) which concentration increases exponentially in plasma until the term of pregnancy. The CD9 ligand has been involved in the production of cytokines contributing to a successful pregnancy [10,37]. According to Abadía-Molina *et al*, the possible role of term decidual leukocytes is related to placental detachment, cytokine production and normal delivery [6]. Thus, the presence of CD9+ dNK cells at term pregnancy could be of physiological relevance.

The precise composition of decidual leukocytes in PE women has remained unclear due to diverse difficulties such as different sources of decidual tissue (placental bed or attached to the placenta) and the various methods used for their characterization (Flow cytometric analysis and immunohistochemistry) [20]. The Flow cytometric analysis used in this study overcomes this problem providing a better quantification method that can manage a high cell number. However, some discrepancies have been found using this method. In the present study the overall amount of each cell population was quantified, while other studies previously counted CD45 leukocytes. Wilczyński *et al *reported an increase in the cytotoxic CD56^+^CD16^+ ^population in the PE group as compared with the control [38], while Rieger *et al *found a decrease in the same population [19]. In contrast, our work did not show any differences between the control and PE groups and we found the cytotoxic population only in a small fraction of the total CD56^+ ^cells. The cytokine-producer CD56^+^CD16^- ^cells constituted the predominant subset observed in this study, as it had been reported during early pregnancy [14].

In our study, the observation of a decreased percentage of CD3^+ ^cells in the PE group was an unexpected finding. During early pregnancy, T cell lymphocytes have been found in a minor leukocyte proportion in decidua [30] and they predominate in the third trimester of gestation, representing 50% to 60% of total leukocytes in this tissue [2]. Our finding of reduced CD3^+ ^cells in patients with PE is in agreement with recent reports of decreased CD8^+ ^cell numbers [19] and T regulatory cells [39], which suggests a possible role of T cells in PE but requires further characterization.

Regarding NK cell function, it has been well established that in addition to the total number of cells, NK cell activity results from a balance between activating and inhibitory receptors. Our results of KIR gene frequencies are in accordance with previous studies of KIR frequencies in Mexican population [40,41]. In the present analysis we found a lower frequency of *KIR2DL2 *gene that could suggest a predisposing effect for PE. However, this difference was not statistically significant. Interestingly, similar results have been reported in recurrent miscarriage [42].

When the repertory of KIR genes was analyzed, we found an increased percentage distribution of healthy women with an activating genotype. Hiby *et al*, found a strong association between the inhibitory genotype (*AA*) and the prevalence of PE and suggested that the activating genotypes protect to PE [25]. Although these genetic data support the notion that dNK cells must be active in order to stimulate the appropriate placental development and differentiation, other researches have found contrasting results in a Japanese population [43].

The widely divergent results obtained from dNK cells at the end of pregnancy raises the necessity to demonstrate their functions and implications in the pathogenesis of PE. Previous work has showed that women with PE have an exaggerated proinflammatory response mediated by pNK cells [44]; therefore it is mandatory to explore the NK1/NK2 response in dNK cells from PE patients. It is known that different factors, such as placental and fetal growth factors, and the local cytokine and hormonal microenvironment modulate the activation of dNK cells throughout gestation. In addition, stromal cells express receptors, ligands, or both, that could interfere with the activity of dNK cells. Importantly, KIRs are also expressed in T cells; e.g., T cytotoxic cells [45]. Moreover KIR expression could change throughout gestation [46].

## Conclusion

In conclusion, we demonstrated the persistence of dNK cells in PE and control women at the third trimester of pregnancy; these dNK cells had a similar phenotype (CD56^+^, CD3^-^, CD16^-^, CD9^+^) to those found during early pregnancy. The persistence of this subset until the end of pregnancy suggests that they may be of physiological relevance throughout pregnancy. The predominance of a KIR inhibitory balance in the PE group could be associated to the physiopathology of PE, but suggests that KIR repertory is not the only determinant factor predisposing to PE.

## List of abbreviations

CI: confidence interval; dNK: decidual NK cells; G: genotype; HLA: histocompatibility leukocyte antigens; KIR: killer cell immunoglobulin like receptors; NK: Natural Killer cells; OR: odds ratio; PBMC: peripheral blood mononuclear cells; PE: preeclampsia; PLGF: placental growth factor; pNK: peripheral NK cells.

## Competing interests

The authors declare that they have no competing interests.

## Authors' contributions

ENSR and MC participated in the design of the study. ENSR performed the experiments, analyzed the data and wrote the manuscript. SNS helped in collecting and processing biological samples. JFRA and CM diagnosed the patients and analyzed the clinical data. The data and the manuscript were analyzed and reviewed by CAMR, EO, JG, AC and MC. All authors read and approved the final manuscript.
